# Comparison of the effect of Dapagliflozin and Pioglitazone on the risk of osteoporosis in postmenopausal women with Type-2 diabetes

**DOI:** 10.12669/pjms.39.5.7580

**Published:** 2023

**Authors:** Osman Son

**Affiliations:** Osman Son, MD Department of Endocrinology and Metabolic Diseases, Anadolu Hospital, Eskisehir, Turkiye

**Keywords:** Osteoporosis, Dapagliflozin, Pioglitazone, Type 2 Diabetes Mellitus

## Abstract

**Objective:**

Type 2 Diabetes mellitus (T2DM) and osteoporosis, which increase with age, are two common diseases with different complications. The risk of fractures due to osteoporosis is 2 to 6 times higher in patients with diabetes mellitus (DM). Medications used in the treatment of DM in addition to the disease itself are associated with the risk of osteoporosis and osteoporotic fractures. This study was planned to examine the effects of pioglitazone and dapagliflozin, used in the treatment of T2DM, on the development of osteoporosis in postmenopausal women.

**Methods:**

This single-centre comparative study was conducted at Endocrine and Metabolic Diseases Polyclinic of a Hospital between April 15, 2019 and April 15, 2020, with a total of 80 postmenopausal female patients with a diagnosis of T2DM and 20 in the control group, aged between 50 and 70. The participants were evaluated under four groups: “Control” without diabetes mellitus (n=20), “Pioglitazone” using (n=30), “Dapagliflozin” using (n=30), and “Other Oral Antidiabetic” using (n=20).

**Results:**

The mean age of the participants was 61.32±6.27 years. There was no statistically significant difference between the groups in the hip and waist T-score values of participants with T2DM in the study (p>0.05). There was no significant difference in waist and hip t-score values between the intervention groups. Pioglitazone and dapagliflozin used in postmenopausal T2DM patients were determined not to make a significant difference in waist and hip bone mineral density values.

**Conclusion:**

Our study revealed that pioglitazone and dapagliflozin can be used in postmenopausal T2DM individuals without known osteoporosis and other osteoporosis risk factors.

## INTRODUCTION

The number of patients with DM is rapidly increasing in all regions of the world. So much that this increase has reached the size of a pandemic. The majority of individuals with diabetes have Type 2 diabetes mellitus (T2DM) (90%). DM cannot be explained only as high blood sugar. It is a chronic metabolic disease with significant morbidity and mortality. Another metabolic disease that is common in all regions of the world is osteoporosis. Osteoporosis is a progressive metabolic bone disease that results in an increase in fracture tendency as a result of low bone mass and deterioration of the microarchitecture of bone tissue.^1^

DM and osteoporosis are two common diseases with different complications. Both T2DM and osteoporosis are diseases that increase with age. This becomes even more important, especially in postmenopausal women. There is a complex relationship between DM and osteoporosis.^2^ The risk of fractures due to osteoporosis is 2 to 6 times higher in patients with diabetes mellitus (DM). Therefore, all individuals with postmenopausal diabetes should be screened for osteoporosis. Although the relationship between DM and osteoporosis has been known for many years, clinical screenings have begun to be performed recently.^3^ Patients with T2DM were determined to have an increased risk of fracture, even though they have higher BMD values than individuals without diabetes. The reason for this is explained by the fact that the risk of falls in individuals with diabetes is higher.^3^,^4^ Bone disorders have increased in both Type1 and T2DM patients, albeit through different mechanisms. Decreased bone strength due to low bone turnover has been identified in both types of diabetes.

Moreover, medications used in the treatment of DM in addition to the disease itself are associated with the risk of osteoporosis and osteoporotic fractures. Factors that increase the risk of osteoporosis and osteoporotic fractures in DM are the accumulation of advanced glycation end products, low bone turnover, and changes in bone microstructure. Furthermore, medications used in the treatment of DM, diabetic peripheral neuropathy, and muscle weakness also contribute to this condition. Drugs used in the treatment of DM such as metformin, sulfonylureas, dipeptidyl peptidase-4 inhibitors (DPP4 inhibitors), insulin, and GLP-1 receptor agonists are noted to be better in terms of osteoporosis.^4^ It has been also stated that the use of thiazolidinediones and sodium glucose transporter-2 inhibitors (SGLT-2) should be avoided in individuals with diabetes who are at risk of osteoporosis and related fractures.^4^-^8^ This study was planned to examine the effects of pioglitazone and dapagliflozin, used in the treatment of T2DM, on the development of osteoporosis in postmenopausal women.

## METHODS

The study was carried out in the Endocrine and Metabolic Diseases Polyclinic of a Hospital between April 15, 2019 and April 15, 2020. Postmenopausal women, who were between 50 and 70 years of age, with a diagnosis of T2DM, who visited the Endocrine and Metabolic Diseases Polyclinic between 15.04.2019 and 15.04.2020, were followed up in the clinic for at least one year were included in the study.

Women under the age of 50 and over the age of 70, who were not diagnosed with T2DM, who did not enter menopause, who had previously been diagnosed with osteoporosis and who had been going through the treatment process due to this diagnosis were excluded from the study. Patients with chronic bowel disease (such as Crohn’s, ulcerative colitis, celiac) despite being in the postmenopausal period with a diagnosis of T2DM, and patients using medications that may cause secondary osteoporosis such as coumadin and steroids were also excluded from the study.

Patients who were included in the study were divided into three groups, 30 of whom were using pioglitazone, 30 of them dapagliflozin, and the other 20 who received medical treatment for diabetes other than these two medications for the last year. Furthermore, 20 non-diabetic postmenopausal female patients over 50 years of age were included in the study as the control group. It was a single-centre comparative study. The initial weight and height of the participants were measured and their BMI values were calculated. Glucose, Creatinine, ALT, GGT, TSH, HbA1C, Calcium, Phosphorus, Vitamin D, Parathorman, insulin resistance, Bone mineral density, waist and hip T-score (BMD) measurements were performed for the participants and the duration of their diabetes was noted. After the parameters were collected, the data of the three groups (those using pioglitazone, dapagliflozin, and other oral antidiabetic drugs) were compared among themselves and with the control group.

### Ethical Approval

This retrospective study was approved by a Health Sciences University Ethics Committee (2019/108) and written informed consent was obtained from all participants. The study was conducted in accordance with the principles of good clinical practice and the 1964 Declaration of Helsinki and its subsequent amendments.

### BMD Measurements

Lumbar vertebra (L1–L4), spine bone mineral density, and femoral neck t-score values were measured using dual-energy x-ray absorptiometry (Hologic, Inc., Bedford).

### Determination of Blood Biochemical Parameters

Blood samples were taken for the measurements of Glucose, Creatinine, ALT, GGT, TSH, HbA1C, Calcium, Phosphorus, Vitamin D, Parathormone and insulin. Analysis of the samples was carried out with a Hitachi 7180 biochemistry automatic analyzer (Hitachi, Tokyo, Japan) in the same Hospital.

### Statistical Analyses

The IBM SPSS Statistics 22 program was used for statistical analysis of the findings obtained in the study. The suitability of the parameters to the normal distribution was evaluated with the Kolmogorov-Smirnov and Shapiro-Wilks tests. During the evaluation of study data, in the comparison of quantitative data, the One-way ANOVA test was used for the comparison of normally distributed parameters between groups, and the Tukey HDS test was used to determine the group that the difference emerged from in addition to descriptive statistical methods (mean, standard deviation, median). Kruskal Wallis test was used for the comparison of the parameters that did not show normal distribution, and Dunn’s test to determine the group that caused the difference. Significance was evaluated at the p<0.05 level.

## RESULTS

The study was conducted with a total of 100 participants including 80 postmenopausal female patients with a diagnosis of T2DM and 20 in the control group, aged between 50 and 70. The mean age of the participants was 61.32±6.27 years. The participants were evaluated under four groups: “Control” without diabetes mellitus (n=20), “Pioglitazone” using (n=30), “Dapagliflozin” using (n=30), and “Other Oral Antidiabetic” using (n=20). There was no statistically significant difference between the groups in terms of mean age, duration of diabetes, and systolic and diastolic blood pressure values (p>0.05) (Table-I).

**Table-I:** Evaluation of the groups in terms of demographic characteristics

	Pioglitazone	Dapagliflozin	Other Oral Antidiabetic	Control	p

	Median±SD	Median±SD	Median±SD	Median±SD	
Age	60.37±5.99 (62)	61.13±6.3 (62.5)	62.05±7.34 (63)	62.3±5.72 (62.5)	0.609
DM (year)	9.63±3.41 (9)	11.47±3.49 (12)	10.6±4.35 (9.5)	-	0.131
SBP	123.5±12.54 (120)	123±14 (120)	124±10.71 (122.5)	120.75±11.27 (120)	0.847
DBP	74±6.75 (70)	77.63±6.75 (80)	73.75±7.05 (72.5)	73.75±7.23 (72.5)	0.156

Kruskal-Wallis Test; SBP: Systolic Blood Pressure; DBP: DiastolicBlood Pressure; DM: Diabetes Mellitus.

There was a statistically significant difference between the groups regarding mean BMI values (p:0.001; p<0.05). According to the post hoc Tukey HSD test performed to determine which group the significance originated from, the Dapagliflozin group was found to have statistically significant higher mean BMI values than the Other Oral Antidiabetic (p:0.020) and Control (p:0.001) groups (p<0.05). There was no statistically significant difference between the other groups (p>0.05)(Table-II).

**Table-II:** Evaluation of the groups in terms of osteoporosis parameters

	Pioglitazone	Dapagliflozin	Other Oral Antidiabetic	Control	p

	Median±SD	Median±SD	Median±SD	Median±SD	
BMI	30.63±5.32 (29.7)^ab^	33.15±5.7 (33.3)^a^	28.81±4.11 (29.2)^b^	27.17±4.53 (27.6)^b^	^+^0.001*
Hip T score	-1.33±0.88 (-1.5)^a^	-1.17±0.76 (-1.3)^a^	-1.04±0.88 (-0.7)^a^	-1.01±0.65 (-0.9)^a^	0.577
Waist T score	-1.35±0.81 (-1.3)^a^	-1.58±0.98 (-1.8)^a^	-1.29±1.1 (-0.9)^a^	-1.03±0.64 (-0.9)^a^	0.158
Calcium	9.48±0.39 (9.5)^a^	9.36±0.56 (9.4)^a^	9.28±0.31 (9.2)^a^	9.34±0.37 (9.3)^a^	0.101
Phosphorus	3.97±0.48 (4)^a^	3.65±0.77 (3.8)^ab^	3.48±0.75 (3.5)^ab^	3.35±0.7 (3.4)^b^	^+^0.009*
Parathormone	58.4±13.77 (62)^a^	52.88±17.69 (56.5)^ab^	60.4±18.27 (65.5)^a^	47.4±12.02 (48.5)^b^	0.013*
Vitamin D	25.15±13.75 (26)^a^	25.57±17.24 (20.5)^a^	22.88±17.29 (22)^a^	25.45±16.76 (23)^a^	0.829
HOMA-IR	4.41±2.51 (4.1)^a^	4.12±1.65 (3.9)^a^	3.82±1.3 (3.7)^a^	3.07±0.79 (3.1)^a^	^+^0.073
HbA1c	7.5±1.72 (6.8)^a^	8.07±1.87 (7.4)^a^	7.81±1.56 (7.3)^a^	-	0.260

Kruskal-Wallis Test^+^One-way ANOVA Test

* p<0.05, HOMA-IR: Homeostatic model assessment- Insulin resistance

***NOTE:*** Different letters in the lines indicate the difference between groups.

There was a statistically significant difference between the groups regarding parathormone levels (p:0.013 ; p<0.05). According to the post-hoc Dunn’s test performed to determine which group the significance originated from, the Control group was found to have statistically significant lower parathormone levels than the Pioglitazone (p:0.008) and Other Oral Antidiabetic (p:0.003) groups (p<0.05). There was no statistically significant difference between the other groups (p>0.05).There was no statistically significant difference between the groups in hip T-score values of individuals with DM who participated in the study (p>0.05). There was no statistically significant difference between the groups in the waist T-score values of individuals with DM who participated in the study (p>0.05).Table-II

There was no statistically significant difference between the groups regarding laboratory parameter values in diabetic individuals (p>0.05) (Table-III).

**Table-III:** Evaluation of the groups in terms of laboratory parameters.

	Pioglitazone	Dapagliflozin	Other Oral Antidiabetic	Control	p

	Median±SD	Median±SD	Median±SD	Median±SD	
Glucose	162.47±59.27 (138.5)^a^	164.67±62.15 (145.5)^a^	162.1±49.95 (149)^a^	85.75±7.87 (86.5)^b^	0.001*
Creatinine	0.73±0.2 (0.7)^a^	0.68±0.17 (0.7)^a^	0.77±0.18 (0.8)^a^	0.35±0.64 (0.6)^a^	0.096
ALT	20.07±7.09 (17)a	18.23±7.36 (16)^a^	20.3±8.14 (17)^a^	19.55±6.2 (19)^a^	0.591
GGT	24.07±10.46 (22)^a^	25.47±14.81 (20,5)^a^	25.3±10.48 (23)^a^	33.2±9.77 (34.5)^b^	0.023*
GFR	90.77±14.74 (95.5)^a^	91.57±10.74 (93.5)^a^	92.5±10.95 (96.5)^a^	93.4±7.02 (92.5)^a^	0.945
TSH	1.7±0.95 (1.5)^a^	1.77±1.1 (1.4)^a^	1.81±1.04 (1.4)^a^	1.23±0.57 (1.2)^a^	0.242

ALT: Alanine transaminase, GGT: Gamma-glutamyl transferase, GFR: Glomerular filtration rate, TSH: Thyroid stimulating hormone Kruskal-Wallis Test

* p<0.05,

***NOTE:*** Different letters in the lines indicate the difference between groups.

## DISCUSSION

The present study finds no significant differences in waist and hipbone mineral density values in postmenopausal T2DM patients using Dapagliflozin and Pioglitazone. DM is one of the most important public health problems today. There are known and more important complications of diabetes. However, another issue that should not be overlooked in individuals with diabetes is osteoporosis and osteoporotic fractures. In the same age group, the risk of fracture in individuals with diabetes is two to six times higher than in those without diabetes.^1^ DM and osteoporosis are two common diseases with different complications.^2^ Furthermore, age and postmenopausal period in women are important factors in the development of osteoporosis. About 30% of all postmenopausal women are affected by osteoporosis.^3^ Although the relationship between DM and osteoporosis has been known for about half a century, this relationship has begun to be further investigated in the last 10-15 years. Osteoporosis due to diabetes is caused by chronic hyperglycemia, advanced glycated end products, and oxidative stress.^3^-^8^

In a study, significant differences were observed in BMD values in the lumbar region between individuals with and without T2DM, but no significant difference was found in the femoral neck region.^9^ Also in the Rotterdam study, higher BMD values were found in the femoral neck and lumbar spine in people with T2DM compared to non-diabetic individuals.^10^ In our study, although the waist and hip t-score values of the diabetic group in postmenopausal women were slightly lower than in the control group, there was no statistically significant difference between them. There was also no significant difference in waist and hip t-score values among the pioglitazone, dapagliflozin, and other oral antidiabetic groups. Moreover, parathormone levels were found to be higher in the diabetic group using pioglitazone and other oral antidiabetic medications compared to the control group. A healthy diet and physical exercise are essential in the prevention and treatment of both diabetes and osteoporosis.^11^

However, the drugs to be chosen for the treatment of diabetes in postmenopausal women with diabetes of a certain age are also important. Drugs used in these patients such as metformin, sulfonylureas, dipeptidyl peptidase-4 inhibitors (DPP4 inhibitors), insulin, and GLP-1 receptor agonists are noted to be better in terms of osteoporosis. It has been stated that insulin can be used in a selected patient group.^12^ However, it has been mentioned that thiazolidinediones should not be preferred.^12^ On the other hand, there is no clear information regarding sodium-dependent glucose transporter two inhibitors (SGLT2I).^12^,^13^ Some studies have noted that it should not be preferred in patients with osteoporosis.^12^-^17^

In our study, when we looked at the effects of these two drugs (pioglitazone and dapagliflozin) on osteoporosis in postmenopausal female patients with T2DM, no significant difference was found in the waist and hip t scores compared to other oral antidiabetics. Obesity is widespread in individuals with T2DM. Looking at the pathophysiology of osteoporosis and fracture risk development, obesity is associated with the prevention of osteoporosis, especially in T2DM.^18^ In our study, the BMI values of the patients using dapagliflozin and pioglitazone were found to be higher than the patients in the control group and the patients using other oral antidiabetic drugs. The reason for pioglitazone (hepatosteatosis) and dapagliflozin (less hypoglycemia, weight loss, and cardiac-positive effects) to be preferred in obese Type-2 diabetic individuals was due to insulin resistance. Furthermore, patients with diabetes have an increased risk of fractures in the humerus, tibia, and ankle regions, apart from the classical waist and hip fracture risk.^19^ One of the limitations of our study was the lack of data on these regions.

The relationship between osteoporosis and fracture risk and HBA1c in individuals with T2DM was investigated. In these studies, no significant relationship was found between HbA1c and lumbar BMD in patients with T2DM. However, a positive correlation was found between the incidence of hip fracture and HbA1. It was stated that more studies are needed to determine whether there is a relationship between HBA1c and BMD or fracture risk.^20^ No significant difference was found between HBA1c and t- scores in our study. It is widely accepted that the risk of osteoporosis increases with the prolongation of the duration of DM disease.^20^ BMD was reported to decrease with the prolongation of the duration of DM, which was supported by several previous studies.^21^Pathological changes accompanying diabetes are as follows: Decrease in bone collagen, decrease in maturation and transformation of bone matrix, and decrease in BMD value as a result of serum calcium deficiency.^22^

In our study, there was no significant difference between the groups in terms of the duration of diabetes. The average duration of DM is between 9 and 12 years. Therefore, no significant difference was found in BMD values between the groups. Vitamin D level is another important factor in the development of postmenopausal osteoporosis.^20^-^22^ In our study, serum vitamin D levels were found to be low in all groups including the control group and the three diabetic groups. Adequate vitamin D intake should be encouraged for the prevention of osteoporosis in individuals with T2DM. In another study, those using insulin among T2DM patients were reported to have higher lumbar BMD values compared to those using only oral antidiabetic.^20^

Some studies in the literature have noted that insulin has an important role in preserving bone mass and preventing bone mass loss as well as controlling blood sugar and that there might be a relationship between osteoporosis and reduced insulin and insulin resistance.^20^-^24^ In our study, there were no significant differences between insulin and insulin resistance between the groups. Therefore, no significant differences were found in waist and hip BMD values. Furthermore, there is a relationship between calcium, phosphorus, parathormone, and vitamin D levels and the risk of osteoporosis and fracture. In our study, parathormone levels among these parameters were higher in the group using pioglitazone and other oral anti-diabetic drugs. Despite this, there was no significant difference in BMD values in each group.

### Limitations

It included sample size was small and other measurement methods related to fracture risk have not been used. There is a need to conduct studies with a bigger sample size.

## CONCLUSION

No significant difference was found in the waist and hip BMD values of the groups of pioglitazone and dapagliflozin, used in postmenopausal T2DM patients, which are noted to increase the risk of osteoporosis and fracture, compared to patients using other antidiabetic drugs and nondiabetic patients. However, other parameters besides the waist and hip BMD values should be taken into account to evaluate the risk of osteoporosis and fracture in T2DM postmenopausal patients. Our study is important in terms of showing that pioglitazone and dapagliflozin can be used in postmenopausal T2DM individuals without known osteoporosis.
